# Advances in local field potential research in prolonged disorders of consciousness: a narrative review

**DOI:** 10.1186/s41016-026-00441-x

**Published:** 2026-07-13

**Authors:** Zhuoyang Li, Tianqing Cao, Jiwei Wang, Sipeng Zhu, Yitong Jia, Kefei Wan, Conghui Li, Yi Yang

**Affiliations:** 1 Department of Neurosurgery, The First Hospital of Hebei Medical University, Shijiazhuang, 050031 China; 2 Department of Neurosurgery, Aviation General Hospital, Beijing, 100012 China; 3 Department of Neurosurgery, Beijing Tiantan Hospital, Capital Medical University, Beijing, China; 4 China National Clinical Research Center for Neurological Diseases, Beijing, China; 5 Chinese Institute for Brain Research, Beijing, China; 6 Beijing Institute of Brain Disorders, Beijing, China

**Keywords:** Prolonged disorders of consciousness, Local field potentials, Central thalamus, Thalamocortical circuits, Deep brain stimulation, Mesocircuit model

## Abstract

Prolonged disorders of consciousness (pDOC)—including coma, vegetative state/unresponsive wakefulness syndrome (VS/UWS), and minimally conscious state (MCS)—remain diagnostically and therapeutically challenging. Even with validated instruments such as the Coma Recovery Scale–Revised (CRS-R), behavioral assessment yields misdiagnosis rates approaching 40%, as fluctuating arousal, motor impairment, and sedation can mask preserved cognition. Because the VS/UWS–MCS distinction directly shapes prognostic counseling, rehabilitation, and decisions about life-sustaining treatment, and because the circuit-level pathophysiology of pDOC is still incompletely mapped, objective physiology-based biomarkers are urgently needed to complement bedside examination. This narrative review, informed by a structured search of PubMed, Embase, MEDLINE, and Web of Science, focuses on studies directly reporting intracranial electrophysiology—particularly local field potential (LFP) recordings from the central thalamus—in patients with pDOC. Indirect evidence from anesthesia paradigms, animal models, and related neuromodulation settings is invoked only to clarify mechanism or methodology. We group current findings into four complementary signal domains: oscillatory activity (slow oscillations and burst suppression through theta, alpha, beta, and gamma rhythms); thalamocortical and corticostriatal functional connectivity; aperiodic/scale-free dynamics reflecting excitation–inhibition balance; and nonlinear complexity measures indexing information integration. Single-unit firing, cross-frequency coupling (CFC), and evoked or perturbational responses are considered alongside these. All four are interpreted within a unifying mesocircuit-and-criticality framework, in which impaired consciousness reflects reduced arousal gain, degraded thalamocortical integration, and departure from the critical dynamical regime sustaining conscious-capable dynamics. Intracranial LFP recordings thus offer a rare mechanistic window onto thalamocortical dysfunction in pDOC and may eventually inform patient stratification, target selection, and mechanism-guided neuromodulation. The evidence base remains limited; however, small samples, etiological heterogeneity, variable recording conditions, sparse direct human data, and a predominance of group- rather than individual-level analyses constrain current inference. Translation is further complicated by unresolved ethical issues around surrogate consent, equitable access, and disclosure of diagnostic uncertainty. Meaningful progress will depend less on technical novelty than on standardized protocols, multicenter validation, and rigorous ethical frameworks.

**Supplementary Information:**

The online version contains supplementary material available at 10.1186/s41016-026-00441-x.

## Background

Prolonged disorders of consciousness (pDOC) describe a spectrum of states in which patients fail to show reliable evidence of awareness after severe brain injury. Coma denotes the absence of both wakefulness and awareness; VS/UWS is distinguished by the return of eye opening and sleep–wake cycles without reproducible behavioral signs of awareness; MCS is defined by inconsistent but discernible behavioral evidence of awareness—such as visual pursuit, localization to noxious stimuli, or command following—and is subdivided into MCS − (non-language-mediated) and MCS + (language-mediated) responses [1]. Classification shapes prognostic counseling, rehabilitation, resource allocation, and decisions about life-sustaining treatment [2], yet even with validated tools such as the CRS-R, misdiagnosis rates reach 40% in some cohorts because fluctuating arousal, motor impairment, aphasia, and sedation can mask preserved cognition [3]. The recognition of cognitive motor dissociation—neural evidence of command following despite absent behavioral response—further exposes the limits of bedside examination and reinforces the case for physiology-based biomarkers.

Non-invasive modalities—scalp EEG, fMRI, PET, and TMS-EEG—remain indispensable for detecting covert cognition and quantifying cortical complexity [4], yet are constrained by volume-conduction smearing in EEG and by temporal resolution insufficient to capture fast oscillatory dynamics and burst–tonic transitions in deep midline structures on neuroimaging [5]. The thalamocortical circuits gating conscious states rely on rapid reciprocal interactions between specific thalamic nuclei and cortical layers that can only be directly observed through intracranial recording. Local field potentials (LFPs) from DBS electrodes in the centromedian–parafascicular (CM-Pf) complex or adjacent intralaminar nuclei capture aggregate synaptic activity within millimeters at millisecond resolution [6], and—paired with multi-unit activity (MUA) recordings—allow thalamic firing patterns to be related to consciousness level [7]. Though restricted to surgical candidates, these recordings provide ground-truth data for calibrating non-invasive biomarkers and elucidating mechanistic substrates of consciousness [8].

The pathophysiological account most widely invoked for pDOC is the anterior forebrain mesocircuit model: consciousness depends on sustained excitatory drive from the central thalamus to frontal cortex and striatum, with the striatum modulating pallidal inhibition of the thalamus to sustain forebrain activation. Diffuse or multifocal injury reduces excitatory input—via structural damage or downstream disfacilitation—producing tonic pallidal over-inhibition, collapse of thalamocortical and corticostriatal signaling, and a global narrowing of neocortical dynamic range that manifests as impaired consciousness. Non-human primate work indicates that consciousness specifically depends on integration across parietal cortex, striatum, and intralaminar thalamus, with deep cortical layers and subcortical structures contributing more to differentiating conscious states than frontal cortex alone [9, 10]. The non-specific matrix thalamic system, projecting diffusely to superficial cortical layers, has emerged as a key modulator of cortical information-processing modes associated with awareness [11]. Together, these lines of evidence frame pDOC as a disorder of circuit-level activation, coordination, and integration across thalamocortical, corticostriatal, and brainstem arousal systems—rather than as an abnormality of any single frequency band or anatomical site.

**Fig. 1:**
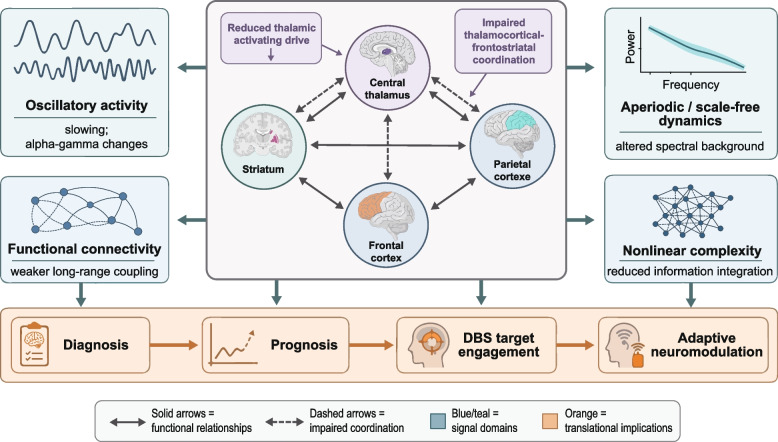
Circuit framework linking LFP domains to pDOC pathophysiology. The schematic illustrates a mesocircuit centered on the central thalamus and its interactions with the striatum, parietal cortex, and frontal cortex. Reduced thalamic activating drive and impaired thalamocortical-frontostriatal coordination are proposed to underlie prolonged disorders of consciousness. Four complementary signal domains are depicted as downstream electrophysiological readouts of this circuit dysfunction: oscillatory activity, functional connectivity, aperiodic/scale-free dynamics, and nonlinear complexity. Characteristic abnormalities include oscillatory slowing, weaker long-range coupling, altered spectral background, and reduced information integration. The lower translational band highlights the potential relevance of these signal domains for clinical applications, including diagnosis, prognosis, DBS target engagement, and future adaptive neuromodulation. This figure emphasizes that different LFP-derived features should be interpreted as complementary indicators of a shared mesocircuit dysfunction rather than isolated biomarkers

We searched PubMed, Embase, MEDLINE, and Web of Science using terms related to pDOC, local field potentials, thalamic recordings, and deep brain stimulation. Priority was given to studies directly reporting intracranial electrophysiology in pDOC, with selected supportive evidence from anesthesia, animal models, and related neuromodulation fields invoked where relevant. Our aims are to synthesize current evidence on intracranial LFP features in pDOC, organize findings within a circuit-based framework, weigh translational implications and limitations, and outline priorities for future research (see Fig. 1).

## Results

### Intracranial LFP features in pDOC

#### Circuit framework for interpreting LFP findings in pDOC

A circuit-level framework is essential for interpreting the diverse electrophysiological features observed in pDOC. The mesocircuit model, proposed by Schiff, provides the most widely cited account of how widespread brain injury produces sustained impairment of consciousness [1]. Severe injuries of any etiology—traumatic, vascular, or hypoxic-ischemic—cause diffuse neuronal loss and axonal damage that reduce excitatory output from the central thalamus, particularly intralaminar and paralaminar nuclei, to frontal cortex and striatum. Because the striatum normally exerts tonic inhibition on the globus pallidus internus, diminished striatal drive leads to excessive pallidal inhibition of the thalamus, creating a self-reinforcing cycle of reduced thalamocortical activation [4]. The result is global down-regulation of anterior forebrain activity that compromises arousal, executive function, and organized behavior.

This framework is enriched by broader thalamocortical models. The matrix–core distinction—diffusely projecting matrix cells modulating widespread cortical excitability while focal core projections support specific sensory relays—explains why central thalamic dysfunction disproportionately disrupts global cortical integration [10]. Computational modeling shows that selective stimulation of high-matrix thalamic regions restores wake-like cortical dynamics including frontoparietal coherence and complex oscillatory patterns, whereas low-matrix stimulation does not [11]. Matrix-enriched nuclei thus appear critical for sustaining conscious states, constituting a shared pathophysiological substrate across heterogeneous pDOC etiologies. Ascending arousal systems—brainstem cholinergic, noradrenergic, and glutamatergic projections—supply the background excitatory drive required for thalamocortical function [9]; damage to these pathways compounds the mesocircuit deficit, helping explain why clinically similar states arise from structurally distinct injuries. A comprehensive summary of LFP studies in pDOC, including patient cohorts, recording targets, signal features, and key findings, is provided in Additional file 1.

#### Oscillatory activity

Neural oscillations recorded intracranially reflect rhythmic discharge of local neuronal populations and their long-range synchronization. In consciousness research these rhythms divide into two functionally distinct regimes. Low-frequency activity—delta (0.5–4 Hz), theta (4–8 Hz), and slow oscillations (< 1 Hz)—predominates when thalamocortical neurons enter burst-firing mode, associated with reduced cortical excitability, sensory gating, and diminished responsiveness. Higher-frequency activity—alpha (8–13 Hz), beta (13–30 Hz), and gamma (> 30 Hz)—emerges under tonic depolarization, supporting recurrent corticothalamocortical interactions underlying sensory binding, attention, and volitional behavior [12]. As excitatory drive to central thalamic neurons diminishes through deafferentation or disfacilitation, the spectral center of gravity shifts toward low frequencies and capacity for organized higher-frequency processing is progressively lost.

The earliest intracranial recordings in pDOC showed spectral profiles dominated by power below 8 Hz, with theta-range spindle-like bursts of 2–5 s in VS/UWS patients during intraoperative microelectrode mapping [6]. A nascent 10 Hz peak was more discernible in an MCS patient than in VS/UWS, becoming considerably more prominent in postoperative anesthesia-free recordings—a shift paralleling that patient’s favorable trajectory. Larger datasets reinforce this pattern. He et al. (2023) recorded MUA and LFPs from the CM-Pf complex in 69 pDOC patients and found normalized alpha-band power significantly higher in MCS than VS/UWS [7]. Stronger alpha and more stable theta at surgery predicted better outcomes, positioning central thalamic oscillations as candidate intermediary variables linking demographics, etiology, and injury duration to consciousness outcomes. Zhang et al. (2025) extended these observations by showing that central thalamic LFP power spectra differentiated VS/UWS from MCS and subsequent recoverers from non-recoverers, with preserved alpha and beta serving as favorable prognostic indicators [8].

Single-case work adds a complementary observation: emotionally salient stimulation can transiently modulate thalamic oscillations even in chronic DoC. Wojtecki et al. (2014) recorded from bilateral central thalamic DBS electrodes and found that familiar emotional speech—versus unfamiliar neutral speech—elicited a right-lateralized beta increase within 1 s and a theta enhancement around 2–3 s [13]. These changes were accompanied by increased thalamocortical theta coherence and local theta–gamma phase–amplitude coupling, suggesting cross-frequency interactions relevant to cognitive-emotional processing can remain partially operative in chronic DoC [14]. Intraoperative mapping in five DoC patients further showed thalamic LFP spectra differ qualitatively from movement disorder patients: DoC patients had higher relative power below 7 Hz and above 30 Hz, whereas movement disorder patients showed stronger 7–30 Hz activity—consistent with loss of the intermediate-frequency regime associated with organized thalamocortical function [15].

Anesthesia paradigms provide a useful mechanistic reference frame, though extrapolation to pDOC requires caution. In non-human primates, propofol-induced unconsciousness produces sustained increases in prefrontal and temporal slow-delta and low-beta/alpha power with reduced posterior beta, while spike rates fall to roughly half waking values; gamma-frequency thalamic stimulation partially reverses this reorganization, reinstating wake-like alpha and gamma in deep cortical layers and central lateral (CL) thalamus when arousal-producing [12]. In humans, propofol disrupts posterior sensory alpha while a novel frontal alpha emerges, each network mapping onto anatomically distinct thalamocortical tracts; the emergent frontal alpha imposes a pathological frequency regime on prefrontal circuits normally operating at beta and gamma during wakefulness [16]. These observations indicate that the shift from high- to low-frequency dominance tracks functional engagement of thalamocortical circuits rather than pharmacological action per se—a principle that may extend to pDOC. The extrapolation has clear limits: anesthesia acts on specific receptors in an otherwise intact brain, whereas pDOC involves structural damage, neuroplastic reorganization, and metabolic derangements that may produce superficially similar but mechanistically distinct signatures.

Several methodological considerations constrain conclusions. Electrode targets vary substantially—CM-Pf complex [7], internal medullary lamina and reticular nucleus [13], CL nucleus [8], and medial thalamus more broadly [5]—and these nuclei occupy distinct positions within the thalamic connectome, so between-study differences may reflect recording geometry as much as pathophysiology. Anesthesia during recording remains a pervasive confound: several key datasets were acquired intraoperatively under general anesthesia [6, 15], and within-study comparisons mitigate but cannot eliminate this issue, as the few paired anesthetized–awake recordings in the same patient reveal substantial spectral reorganization upon anesthesia withdrawal [6]. Stimulation artifacts persist despite bipolar re-referencing, particularly where harmonics overlap with gamma activity. Volume conduction from internal capsule, caudate, and adjacent cortex may further contaminate signals; imaginary coherence and bipolar derivations attenuate but do not eliminate this effect. Finally, the evidence base is dominated by case reports and small series, with only two studies to date exceeding 20 patients [7, 8]. Oscillatory abnormalities in central thalamic LFPs are thus best interpreted as state-associated correlates of thalamocortical dysfunction rather than stand-alone diagnostic biomarkers.

#### Functional connectivity

Consciousness depends on coordinated information exchange across distributed neural populations rather than any single region. Functional connectivity—the statistical dependence between signals from spatially separated sites—indexes this coordination across scales, from local microcircuit coupling to long-range thalamocortical coherence. The mesocircuit model casts the central thalamus as a relay sustaining recurrent excitatory loops with frontal cortex and striatum, and disruption of these loops is predicted to produce the graded loss of behavioral responsiveness seen across the DoC spectrum [4]. Intracranial recordings offer a privileged vantage point for assessing this connectivity, bypassing scalp-EEG spatial blurring and enabling phase, coherence, cross-frequency, and directed-information measurements at millisecond precision. Their trade-off is that electrode coverage follows clinical targeting rather than experimental design, rendering connectivity estimates focal and potentially unrepresentative of broader network architecture.

The most direct evidence for disrupted thalamocortical communication in pDOC comes from simultaneous recordings of thalamic single-unit activity alongside cortical field potentials or scalp EEG. Magrassi et al. (2018) recorded single neurons from the CM-Pf, CL, and paralaminar mediodorsal nuclei in six DoC patients and computed thalamocortical cross-correlation functions (TCCFC) between thalamic spike trains and homolateral parietal cortical activity [5]. Significant TCCFC—indicating preserved functional monosynaptic or oligosynaptic linkage—was identified exclusively in MCS patients, with short-latency peaks (< 3 ms) consistent with intact feedforward and feedback pathways. VS/UWS patients showed no significant thalamocortical cross-correlations despite spontaneously active thalamic neurons, suggesting that disconnection between thalamic firing and cortical dynamics is more fundamental to the VS/UWS state than any absence of thalamic discharge per se. Thalamic neurons firing synchronously with cortical EEG were far more common in MCS than VS/UWS, while bursting neurons—the signature of hyperpolarized thalamic membranes under excessive inhibition—were paradoxically rare in both groups. This profile aligns more closely with disfacilitation arising from widespread excitatory deafferentation than with exaggerated pallidal inhibition, consistent with the mesocircuit model’s emphasis on loss of excitatory drive to the central thalamus as a primary mechanism.

Oscillatory coherence analyses extend this picture. Wojtecki et al. (2014) demonstrated condition-dependent thalamocortical coupling in a chronic DoC patient: theta-band coherence between central thalamic LFPs and parieto-central scalp EEG was significantly enhanced during familiar emotional speech compared with unfamiliar neutral speech, with the imaginary part of coherence deviating from zero, confirming a genuine phase relationship rather than volume conduction [13]. Locally, theta phase was coupled to gamma amplitude selectively during the emotionally salient condition, suggesting the central thalamus retains some capacity for stimulus-driven, frequency-specific cortical communication even in chronic DoC—though strong sensory or emotional input may be required to manifest it. Zhang et al. (2025) provided the most comprehensive characterization of central thalamic connectivity dynamics and recovery to date [8]. Recording from the CM-Pf in 23 patients, they identified theta rhythm stability—the temporal consistency of theta oscillatory power—as the single most informative electrophysiological feature for predicting individual recovery across diverse clinical backgrounds. Computational modeling suggested that theta stability reflects the functional integrity of thalamocortical recurrent loops: stable theta corresponds to a central thalamic state near the threshold for sustained rhythmic engagement with cortex, whereas unstable theta indicates a circuit hovering near the quiescent state of disfacilitation. Theta stability thus functions not merely as a spectral descriptor but as a proxy for the central thalamus’s capacity to maintain the temporally coherent output required for cortical arousal.

These intracranial findings sit within a broader multimodal literature documenting network fragmentation in DoC. TMS-EEG work shows that effective cortical information flow is profoundly suppressed in VS/UWS relative to MCS and healthy controls; Bai et al. (2024) found that frontal and parietal TMS in VS/UWS produced spatially restricted, short-lived information propagation networks with significantly fewer regions in bidirectional exchange, and the interactive ROI rate correlated with clinical consciousness scales [17]. Functional neuroimaging has independently converged on thalamic disconnection from cortical networks—particularly default mode and executive control networks—as a signature feature scaling with severity and predicting recovery. Warren et al. (2025) further showed that the normative connectivity profile of CM-Pf DBS sites predicted clinical improvement: stronger connectivity with the anterior forebrain mesocircuit and brainstem arousal structures was associated with greater likelihood of meaningful recovery, and this network overlapped significantly with circuits disrupted by consciousness-impairing stroke lesions and seizure foci, pointing to convergence across etiologically distinct conditions on a common substrate [18]. In non-human primate intracranial work, Afrasiabi et al. (2021) showed that integrated information (Φ*) tracked stimulation-induced consciousness changes more robustly than power or entropy alone, peaking in parietal deep layers and subcortical structures including thalamus and striatum [9], while Toker et al. (2024) showed that slow-to-fast cross-frequency information transfer between anatomically connected thalamic and cortical regions is present during waking but collapses during propofol anesthesia, tied to loss of criticality in thalamocortical dynamics [19]. Across spike-level coupling, TMS-evoked directed flow, fMRI connectivity, and computational integration measures, the disruption of thalamocortical communication thus appears neither frequency-specific nor spatially confined but a multi-level breakdown of mesocircuit-relevant pathways.

Several methodological considerations temper these conclusions. Connectivity metrics are sensitive to analytic choices—referencing scheme, epoch length, frequency resolution, and algorithm—and different methods on the same data can yield discordant results; the studies reviewed here employ a heterogeneous mix of spike-field cross-correlation [5], wavelet coherence [13], and model-derived theta stability indices [8], making direct quantitative comparison difficult. Apparent coupling need not reflect true interaction: common input, reference contamination, and non-oscillatory broadband transients can inflate connectivity estimates, and although imaginary coherence and bipolar re-referencing attenuate volume conduction, residual coupling may originate from structures along the electrode trajectory rather than the intended thalamic and cortical sources. The direct intracranial evidence base remains small—six, one, and twenty-three patients in the three principal studies—with a single-case report contributing disproportionately to inferences about cross-frequency dynamics. Recording conditions vary between intraoperative anesthesia and postoperative awake states, a confound not easily separated from DoC effects given that anesthetics independently alter thalamocortical connectivity. Finally, normative connectivity analyses used to infer network-level implications of focal DBS sites draw on atlas-based estimates from neurologically intact populations and may not represent the reorganized connectome of severely injured patients. Taken together, while available intracranial evidence consistently implicates disrupted thalamocortical connectivity as a core feature of pDOC, its precise topology, directionality, and prognostic specificity await resolution through larger, methodologically standardized studies employing patient-specific connectivity mapping.

#### Scale-free features

Neural field potentials contain not only periodic oscillations but also an aperiodic component whose power decays with frequency according to an approximate 1/f power law. This component, characterized by its spectral exponent (SE)—the slope of the power spectrum in log–log space—is increasingly recognized as carrying physiologically meaningful information distinct from and complementary to oscillatory measures [20]. Computational and empirical work suggests the exponent reflects the aggregate balance between synaptic excitation and inhibition (E/I balance) within local populations: a steeper (more negative) exponent indicates inhibitory dominance, a flatter exponent relatively greater excitatory tone [21, 22]. This is directly relevant to consciousness, because the mesocircuit model predicts that pDOC involves progressive loss of excitatory drive to central thalamic and frontal circuits—a change that should steepen the aperiodic slope. Accurate estimation requires algorithmic separation of periodic peaks from the 1/f background, formalized by the specparam (formerly FOOOF) framework, since failure to do so risks conflating changes in oscillatory power with changes in aperiodic structure.

Direct intracranial evidence on aperiodic features in pDOC remains sparse but is emerging. Zhao et al. (2025) computed SE from resting-state scalp EEG in 15 DoC patients, 9 conscious brain-injured controls, and 23 healthy controls, finding that narrowband SE (1–20 Hz) differentiated DoC from both control groups and further discriminated MCS from VS/UWS, with more negative exponents indicating lower consciousness levels [23]. SE in this range correlated positively with composite CRS-R scores and visual function subscale ratings, and longitudinal tracking in an individual case revealed progressive flattening of the slope alongside behavioral recovery. The authors interpreted the steepened SE in DoC as a shift of thalamocortical E/I balance toward inhibitory dominance—consistent with the mesocircuit prediction of reduced excitatory throughput—and noted that the 1–20 Hz range outperformed broadband (1–40 Hz) and high-frequency (20–40 Hz) windows, suggesting the aperiodic signature most informative for consciousness resides in the band dominated by thalamocortical dynamics. No study has yet reported specparam-decomposed aperiodic exponents from intracranial thalamic recordings in pDOC, but earlier spectral profiles provide indirect support: central thalamic LFPs in VS/UWS are dominated by low-frequency power with steep spectral roll-off [6, 7]—consistent with a steepened aperiodic exponent—whereas MCS patients show relatively flatter spectra with preserved alpha and beta peaks. As these studies did not formally decompose periodic and aperiodic components, whether reported spectral differences reflect the exponent itself or redistributed oscillatory power remains unresolved.

Convergent but indirect evidence from anesthesia and movement disorder studies establishes that the aperiodic exponent is sensitive to pharmacological and pathological perturbations of subcortical–cortical circuits. In Parkinson’s disease patients undergoing propofol-induced transitions, subthalamic nucleus (STN) LFPs show significant steepening of the aperiodic exponent at loss of responsiveness—a change dissociable from concurrent beta-band alterations and specific to the STN rather than simultaneously recorded frontal EEG [24]—demonstrating that aperiodic features can capture subcortical state changes that oscillatory power alone may miss, within the same cortico-basal ganglia-thalamocortical loop implicated by the mesocircuit model. Mechanistic grounding comes from work linking the STN exponent to E/I balance: levodopa flattens the exponent while the untreated parkinsonian state—characterized by excessive pallidal inhibition—is associated with steeper slopes, and computational models reproduce these effects through GABAergic/glutamatergic manipulation, supporting interpretation of the exponent as an E/I ratio index rather than non-specific noise [22]. Two caveats limit direct extrapolation to pDOC. Aperiodic parameters differ inherently between recording sites: thalamic and STN LFP spectra lack the spectral knee characteristic of cortical electrocorticography, and their exponents are systematically lower, likely reflecting cytoarchitectural, cellular, and current-source differences, so thalamic exponents in DoC patients cannot be directly compared with cortical values under identical parameterization [21]. The estimated exponent is also critically dependent on the frequency range over which it is fitted—different bandwidth windows in the same dataset yield significantly different values and sometimes qualitatively different conclusions about brain state—a dependency particularly problematic in pDOC intracranial recordings, where pathological oscillatory peaks and stimulation harmonics may distort the fit if not carefully excluded [25].

Several further limitations should be acknowledged. No study has applied formal periodic–aperiodic decomposition to thalamic LFPs in DoC; available intracranial evidence relies on visual spectral inspection or traditional band-power analyses that do not isolate the aperiodic component, and the only study explicitly computing SE in DoC used scalp EEG with a moderate sample (*n* = 15). The exponent’s interpretation as an E/I balance index, though supported by computational and Parkinson’s disease pharmacological evidence, has not been validated in the injured brain, where gliosis, axonal degeneration, and chronic pharmacotherapy may unpredictably alter the synaptic-E/I-to-spectral-slope mapping; DBS artifacts and their harmonics may further distort the fit if not rigorously removed. The mapping from aperiodic features to consciousness level may also be non-monotonic: a nonlinear relationship between spectral complexity and propofol sedation depth has been reported, implying that a simple slope metric may not fully capture the underlying structure. Aperiodic spectral features thus represent a promising complement to oscillatory biomarkers, but their utility in pDOC awaits validation through dedicated intracranial studies using standardized decomposition, site-appropriate parameterization, and adequately powered cohorts.

#### Nonlinear dynamical characteristics

Integrated Information Theory (IIT) proposes that consciousness corresponds to a system’s capacity to generate a large repertoire of distinguishable states while maintaining irreducible causal integration across its parts; consciousness is identified with integrated information (Φ), maximal when a system is both highly differentiated and integrated [26]. This motivates empirical measures—entropy, Lempel–Ziv complexity, and the perturbational complexity index (PCI)—that capture these properties from neural signals. PCI, a direct IIT operationalization, quantifies spatiotemporal complexity of TMS-evoked cortical responses and discriminates conscious from unconscious states at the individual level across sleep, anesthesia, and DoC [27]. For intracranial central thalamic recordings, nonlinear dynamical measures offer a complementary perspective, assessing whether the thalamocortical system operates near criticality—the regime IIT identifies as the physical substrate of consciousness.

Direct evidence that integration measures outperform conventional spectral indices comes from primate intracranial work. Afrasiabi et al. (2021) compared entropy (H), mutual information (I), and integrated information (Φ*) across waking, sleep, anesthesia, and thalamic stimulation-induced arousal in macaques, finding that Φ* outperformed all other measures in decoding conscious states and was the only metric tracking fine-scale, stimulation-induced changes in consciousness; the minimum information partitions underlying Φ* consistently grouped parietal deep layers with thalamus and striatum during conscious states, whereas this parietal–subcortical integration broke down during unconsciousness [9]. The result directly implicates integration across the parietal–striatal–thalamic system as a hallmark of consciousness and suggests that integration measures applied to pDOC thalamic recordings may outperform power- or entropy-based approaches.

Complementary human evidence comes from both scalp and intracranial work. Qu et al. (2024) showed that scalp-EEG approximate entropy (ApEn) was systematically lower in VS/UWS than MCS, and that stimulus-induced ApEn changes during preferred music distinguished the two states with over 90% classification accuracy when combined with machine learning [28]. Using thalamic LFPs in 23 DoC patients, Zhang et al. (2025) demonstrated that theta rhythm stability—inversely related to dynamical complexity—was the strongest single predictor of recovery: patients with intermediate stability, consistent with near-critical dynamics, showed favorable outcomes, whereas those locked into excessively stable periodic regimes did not [8]. Mechanistic grounding comes from Toker et al. (2024), who used simultaneous thalamic and cortical LFPs across waking, propofol anesthesia, and spike-and-wave seizures to show that cross-frequency directed information transfer—from slow (1–13 Hz) thalamic to fast (52–104 Hz) cortical activity—was present during waking but collapsed during both anesthesia and seizures [19]. This collapse was not explained by spectral power changes nor captured by broadband transfer entropy, indicating that consciousness depends specifically on cross-frequency information routing rather than mere thalamocortical signal transmission. Waking dynamics operated near the edge of chaos, anesthesia pushed dynamics toward excessive order, and seizures locked them into periodic orbits; a mean-field cortico-basal ganglia-thalamocortical model reproduced these transitions, bridging the mesocircuit model with IIT. Bai et al. (2024) demonstrated that TMS-evoked effective information flow was profoundly diminished in VS/UWS compared with MCS, with the proportion of regions in bidirectional exchange correlating with CRS-R scores [17]; this mirrors the intracranial evidence of collapsed cross-frequency thalamocortical transfer and absent spike-field coupling in unconscious states, converging on the interpretation that consciousness requires not merely thalamocortical connections but their capacity to support complex, integrated, bidirectional exchange.

Several limitations constrain application of complexity and integration measures to pDOC intracranial recordings. No study has yet computed Φ*, PCI, or formal complexity decompositions from thalamic LFPs in human DoC: intracranial integration evidence derives from non-human primates [9], criticality evidence from animal anesthesia and seizure models [19], and human DoC studies have relied on scalp EEG [17, 28]. Whether complexity–criticality–consciousness relationships observed under controlled pharmacological conditions generalize to the chronically injured brain—where structural lesions, gliosis, and plastic reorganization may fundamentally alter dynamical properties—remains untested. Complexity metrics are sensitive to data quality, epoch length, and parameter choices, can yield discordant results across settings, and Φ* computation becomes intractable for high-dimensional systems, requiring approximations of uncertain accuracy. PCI, while clinically validated, primarily reflects cortical dynamics with thalamic contribution inferred rather than directly measured. Recording artifact-free stationary epochs from clinically unstable DoC patients further constrains nonlinear estimate reliability. Dedicated studies combining intracranial thalamic recordings with validated complexity algorithms and clinically meaningful outcomes will be needed to determine whether central thalamic nonlinear dynamics can serve as reliable pDOC biomarkers.

#### Additional electrophysiological features

The preceding sections focused on four major electrophysiological domains: spectral power, functional connectivity, aperiodic features, and nonlinear complexity. Intracranial thalamocortical recordings capture additional signal features mechanistically relevant to consciousness and increasingly documented in animal models, anesthesia paradigms, and emerging human work. Five feature families merit brief consideration.

*Cross-frequency coupling (CFC)*, in which a lower-frequency rhythm’s phase modulates higher-frequency amplitude or timing, provides a candidate mechanism by which long-range thalamocortical coordination organizes local cortical computation. Directed cross-frequency transfer from slow (1–13 Hz) thalamic to fast (52–104 Hz) cortical LFPs is a hallmark of waking in rodents and macaques, collapsing during propofol anesthesia and generalized seizures in a manner not explained by spectral power alone [19]. In humans, Fang et al. (2025) showed that phase–amplitude coupling between high-order thalamic nuclei (mediodorsal and pulvinar) and prefrontal cortex was selectively enhanced during conscious perception of near-threshold visual stimuli, predicted trial-by-trial outcomes, and was diminished during missed trials [29]. CL stimulation during macaque propofol anesthesia restores alpha–gamma thalamocortical coherence coinciding with behavioral arousal [10], and modeling indicates matrix thalamic neurons, via diffuse cortical projections, are uniquely positioned to reinstate cortical processing modes associated with waking, including susceptibility and Kolmogorov complexity measures dependent on cross-frequency structure [11]. Although no study has yet measured CFC directly from thalamic electrodes in DoC patients, this convergent evidence positions thalamocortical CFC as a necessary substrate for conscious access and a high-priority DBS target.

*Burst–tonic firing patterns* in thalamic relay neurons are governed by low-threshold T-type calcium channel de-inactivation. Burst firing predominates during unconscious states (deep sleep, anesthesia) and reduces faithful afferent relay, whereas tonic firing characterizes waking and supports high-fidelity transmission [10]. Propofol anesthesia in macaques shifts CL neurons toward burst firing with reduced spike rates, particularly in deep cortical layers; effective thalamic stimulation reverses this pattern, restoring tonic-like activity, behavioral arousal, alpha–beta coherence, and gamma power [10, 12]. Direct evidence of burst–tonic transitions in human pDOC remains scarce given the rarity of single-unit recordings, but both empirical and computational work predict that DoC involves thalamic neurons locked in a burst-dominated regime due to chronic disfacilitation [11]. Sensing-enabled DBS devices detecting burst-related LFP signatures could provide a clinically feasible proxy for monitoring this firing-mode transition in pDOC.

*Evoked and perturbational responses* address a limitation inherent to resting-state measures, which capture only spontaneous dynamics and may miss networks that are structurally intact but functionally silent—a distinction with direct clinical implications for identifying recovery-potential patients. In anesthetized macaques, thalamic DBS restored not only resting-state spectral and connectivity signatures of consciousness but also cortical responses to auditory novelty—abolished under deep propofol and restored specifically by effective central thalamic stimulation [30]; Bastos et al. (2021) similarly found thalamic stimulation during anesthesia increased both spontaneous cortical firing and stimulus-evoked responses, with evoked recovery preceding the return of spontaneous wake-like patterns [12]. In human DoC, auditory-evoked ApEn changes discriminated VS/UWS from MCS with higher accuracy than resting-state complexity alone [28]. Collectively, these findings indicate that thalamocortical capacity to generate complex evoked responses—rather than merely exhibit resting-state oscillations—may be a more sensitive marker of consciousness-compatible network function, and that combining intracranial thalamic recordings with sensory or direct electrical perturbation could help identify covertly aware patients whose circuits retain stimulus-driven integration capacity despite absent behavioral output.

*Spike-field coupling* indexes how ensemble firing is organized by network rhythms through the temporal relationship between individual spikes and the phase of population-level LFP oscillations. In waking macaques, CL spikes are phase-locked to cortical alpha and gamma in a layer-specific manner—preferentially coupling to deep-layer alpha and superficial-layer gamma, consistent with intralaminar projection patterns—and propofol anesthesia disrupts this coupling, with effective thalamic stimulation partially restoring it [10]. Bastos et al. (2021) further showed that propofol unconsciousness is accompanied by selective loss of gamma-range thalamocortical spike-field coherence, while low-frequency spike-field coupling is preserved or even enhanced—a pattern consistent with burst-dominated firing blocking high-frequency information relay [12]. Human intracranial evidence from Fang et al. (2025) indicates that spike-rate changes in high-order thalamic nuclei during conscious perception are temporally coupled to frontal cortical high-gamma activity via phase transfer entropy, establishing a directed thalamus-to-cortex information flow absent during unconscious processing [29]. No study has yet reported spike-field coupling from thalamic electrodes in pDOC, but sensing-enabled DBS systems can resolve LFP phase dynamics with sufficient precision to estimate spike-field relationships from population-level signals, representing a promising direction for adaptive stimulation.

*Critical dynamics* refer to the hypothesis that conscious brain states operate near a phase transition between ordered and disordered dynamics—a regime maximizing state repertoire, information transmission, and responsiveness to perturbation. Toker et al. (2024) provided the most direct evidence linking thalamocortical criticality to consciousness: waking-state thalamic and cortical LFPs operated near the edge of chaos, whereas anesthesia pushed the system toward supercritical stability and seizures locked it into periodic orbits, with proximity to the edge-of-chaos point predicting cross-frequency information transfer strength [19]. Computational work shows that selective activation of matrix thalamic neurons moves cortical dynamics toward a critical regime characterized by increased susceptibility, participation coefficient, and Kolmogorov complexity—the same dynamical signatures distinguishing waking from anesthesia in empirical data [11]. Direct criticality assessment from pDOC thalamic recordings has not been performed, but Zhang et al. (2025)’s theta stability findings—in which intermediate (near-critical) theta dynamics predicted recovery, whereas excessively stable or irregular patterns did not—provide indirect but compelling support that central thalamic dynamical regime determines recovery potential [8]. Convergence with IIT [26] and PCI [27]—both predicting that conscious brains maximize intrinsic complexity and perturbational responsiveness—suggests criticality-based metrics applied to intracranial thalamic recordings could serve as mechanistically grounded biomarkers for adaptive DBS in pDOC.

#### Integrated interpretation across signal domains

The preceding sections examined oscillatory power, functional connectivity, aperiodic features, nonlinear complexity, and additional electrophysiological signatures as separate analytic domains. These are not independent phenomena but coupled manifestations of the same thalamocortical circuit operating in a particular dynamical regime, organized along four mechanistic axes: arousal gain, network integration, E/I balance, and dynamical state transitions.

The mesocircuit model positions the central thalamus as a gain-modulation hub whose diffuse matrix projections set cortical excitability [4]. When this gain falls, consequences appear simultaneously across signal domains: thalamic neurons shift from tonic to burst firing, generating high-amplitude slow oscillations [10, 12] while degrading the precise spike timing required for thalamocortical phase-locking—explaining why VS/UWS patients can harbor spontaneously active thalamic neurons that show no cortical coupling [5]; the aperiodic exponent steepens as inhibitory tone rises [24]; and complexity falls because burst-dominated dynamics restrict the state repertoire [9, 19]. Reduced alpha–beta power, absent thalamocortical coupling, steepened spectral slope, and diminished complexity thus reflect a single pathophysiological process: loss of thalamic arousal gain. Effective thalamic stimulation reverses these changes in parallel—50 Hz CL stimulation in macaques restores tonic firing, deep-layer cortical spike rates, and alpha–gamma coherence within seconds [10], with simultaneous recovery of cortical firing, thalamocortical synchronization, alpha–beta power, and evoked responses [12]. In human pDOC, DBS-induced theta coherence increases track clinical improvement, with theta stability emerging as the strongest single recovery predictor [8].

Consciousness requires both integration (irreducible interdependence across components) and differentiation (a rich repertoire of distinguishable states) [26], and the reviewed features map onto these dual requirements. Connectivity and CFC metrics—theta thalamocortical coherence, spike-field coupling, and directed cross-frequency information transfer—capture integration; when these collapse, the thalamus becomes an isolated oscillator unable to support conscious processing regardless of local activity, as shown directly by Afrasiabi et al. (2021): Φ* uniquely tracked fine-scale consciousness changes during thalamic stimulation, with minimum information partitions consistently grouping parietal cortex with thalamus and striatum during conscious states [9]. Spectral power, aperiodic exponent, and entropy metrics capture differentiation: loss of alpha–beta peaks, steepened aperiodic slope, and reduced complexity in VS/UWS mark a low-differentiation regime. Zhang et al. (2025) further showed these axes can dissociate in recovery, identifying two distinct subgroups—one with high theta power (restored activation) and another with high theta stability (restored integration dynamics) [8].

E/I balance provides the biophysical mechanism linking features across domains. The mesocircuit model predicts pDOC involves a shift toward inhibitory dominance within the central thalamus, driven by excitatory deafferentation and excessive pallidal inhibition [4]; this shift steepens the aperiodic exponent, pushes oscillatory dynamics toward low-frequency dominance via burst firing, degrades connectivity by disrupting precise spike timing, and confines the system far from criticality. Müller et al. (2023) showed computationally that selective activation of matrix—not core—thalamic neurons simultaneously shifted cortical dynamics toward recovered firing rates, increased functional connectivity, enhanced susceptibility, and higher Kolmogorov complexity [11], and Huang et al. (2024) provided complementary human fMRI evidence that propofol selectively disrupts the functional geometry linking matrix-projecting nuclei to transmodal cortex while sparing core pathways [31]. The critical biophysical variable thus appears to be E/I balance specifically within the matrix thalamocortical system.

Criticality offers the most parsimonious unifying framework. Systems near a phase transition between order and disorder maximize dynamic range, information capacity, and perturbation sensitivity—properties mapping directly onto conscious-state electrophysiology. Toker et al. (2024) showed that waking thalamocortical dynamics operate near the edge of chaos, and that departures in either direction—toward excessive order (anesthesia) or excessive periodicity (seizures)—abolish both consciousness and cross-frequency information transfer [19]. This helps explain why hyper-synchronized seizures and hypo-activated DoC both produce unconsciousness despite opposite spectral profiles: both represent departures from the same narrow critical regime. Indirect human support comes from Zhang et al. (2025): intermediate theta stability predicted recovery while both excessively stable and excessively variable patterns did not—precisely the prediction of a criticality framework in which patients closest to the critical point retain maximal capacity for DBS-induced state transitions [8].

Taken together, diverse electrophysiological features are best understood as complementary projections of the system’s position in dynamical state space relative to a critical point. For clinical translation, composite indices combining power, connectivity, aperiodic slope, and complexity from sensing-enabled DBS devices could estimate this position and serve as control signals for adaptive stimulation. The framework remains largely theoretical for human pDOC; however, no study has simultaneously measured all feature domains from intracranial thalamic recordings in DoC patients, and heterogeneous lesion patterns may produce dissociations that a unified model cannot accommodate without patient-specific calibration.

### Translational implications

#### Diagnostic potential and limitations

Thalamic LFP features hold promise as adjunctive markers for differentiating residual awareness states. He et al. (2023) showed that alpha-band MUA power and spike–LFP synchronization differed significantly between MCS and VS/UWS [7], while Wang et al. (2025) demonstrated that the tonic-to-burst firing ratio graded consciousness across the VS/UWS–MCS–eMCS continuum [32]. Together these observations suggest thalamic electrophysiology may help identify behaviorally unresponsive patients with covert awareness. Any such role is adjunctive rather than stand-alone: invasive implantation restricts applicability to DBS candidates, and inter-patient variability driven by heterogeneous lesions, electrode placement, and the absence of standardized protocols precludes universal diagnostic thresholds. Thalamic LFP features are therefore best interpreted within a comprehensive multimodal assessment.

#### Prognostic stratification

The same signals may help identify patients with recovery potential. Zhang et al. (2025) reported that a composite metric combining theta stability, theta power, and CFC predicted 1-year outcomes (AUC 0.84) and identified two recovery subgroups with distinct neurodynamic profiles [8]; Wang et al. (2025) added that higher preoperative tonic firing predicted MCS emergence after DBS [32]. Clinical-level data reinforce this: Yang et al. (2023) reported markedly higher DBS response rates in MCS (83.3%) than VS/UWS (8.3%) [33], and a 49-patient meta-analysis identified age, time from injury to implantation, and preserved striatal volume as the strongest predictors of DBS responsiveness [34]. Cautious interpretation is warranted: predictive value rests on retrospective DBS cohorts, leaving open whether LFP features predict spontaneous recovery; optimal assessment timing is unknown; and interactions with etiology, lesion pattern, and chronicity require systematic prospective investigation before these markers become clinically actionable.

#### Target engagement and parameter optimization

LFP recordings provide a direct readout of whether DBS engages the target circuit. Stimulation sites producing robust frontal cortical evoked potentials are associated with clinical improvement [18], and precise electrode alignment with the CL/DTTm fiber bundle predicted cognitive improvement in the CENTURY trial [35]. Parameter optimization is harder. Preclinical work shows frequency-specific effects (50 Hz CL stimulation restored cortical dynamics during anesthesia, whereas 10 Hz and 200 Hz did not 10), but clinical translation lags: Bergeron et al. (2025) documented marked heterogeneity across centers (10–200 Hz, 60–450 μs) with no consensus on optimal settings [34], and Dutta et al. (2025) similarly noted that parameter variability precluded definitive conclusions about frequency-dependent efficacy [36]. Using LFP biomarkers such as theta stability to guide personalized programming is theoretically attractive but empirically untested in pDOC.

#### Adaptive neuromodulation as a future direction

The longer-term vision is closed-loop DBS that monitors thalamic LFP features and adjusts stimulation in real time to maintain thalamocortical dynamics near the critical regime associated with consciousness. The rationale is compelling—if consciousness emerges from near-critical dynamics, a control algorithm detecting deviations could apply corrective stimulation. Several obstacles preclude near-term implementation: a reliable real-time control signal must be validated against confounds (movement, medication, circadian fluctuation); the delayed, cumulative nature of DBS effects in DoC—unfolding over hours to days rather than seconds—complicates standard feedback architectures; safety mechanisms must guard against seizure-like induction; and the regulatory pathway remains undefined absent randomized trials demonstrating superiority over conventional management. Overall, current LFP findings have greater value for hypothesis generation, patient stratification, and mechanism-informed trial design than for routine deployment, with translation requiring large prospective multicenter studies using standardized protocols and long-term outcome assessment.

#### Risks, ethics, and non-invasive alternatives

The invasive nature of intracranial recording and DBS in pDOC raises procedural and ethical challenges. Procedural risks—intracranial hemorrhage, infection, hardware malfunction, and seizure induction—are well documented in DBS for movement disorders but carry heightened significance in a population with limited physiological reserve and impaired symptom reporting [34, 37]. The risk–benefit calculus is further complicated by the possibility that partial restoration of awareness without functional independence may be experienced as distressing—a scenario in which increased consciousness paradoxically worsens subjective well-being [37].

Ethical challenges are particularly acute because pDOC patients cannot provide informed consent. Surrogates must authorize invasive procedures on behalf of individuals whose current preferences and experiential states are unknown; recent frameworks emphasize that such consent must balance potential benefit against the vulnerability of a population unable to self-advocate, yet DoC consent forms rarely acknowledge either the limited understanding of subjective experience or population-specific risks [38, 39]. Disclosure of findings—particularly evidence of covert consciousness—can profoundly shape surrogate decisions about life-sustaining treatment, requiring transparent communication of diagnostic and prognostic uncertainty to avoid therapeutic misconception and premature withdrawal of care driven by cognitive biases or incomplete assessment [40, 41]. Access to advanced diagnostics and post-acute rehabilitation is further marked by socioeconomic, geographic, and insurance-related inequities affecting underserved populations [42], and surrogate decision-making is itself shaped by family control-preference variability, the absence of standardized methods for reconstructing incapacitated patients’ preferences, and physician framing effects [43, 44]. Clinical deployment of LFP-based diagnostics or DBS in pDOC must therefore be embedded within rigorous ethical oversight—independent ethics review, structured surrogate consent addressing both potential benefits and the risk of awareness without functional recovery, transparent communication of uncertainty, equitable access frameworks, and ongoing monitoring of neurological and psychological outcomes in patients and families.

Because invasive approaches have inherently limited scalability, non-invasive techniques offer complementary pathways for validation and extension. Scalp EEG, despite lower spatial resolution, can capture cortical signatures of thalamocortical dysfunction—spectral shifts, connectivity alterations, complexity reductions—that parallel the subcortical findings reviewed here, given standardized protocols [45]. TMS-EEG provides a particularly informative non-invasive probe: TMS-evoked effective information flow is markedly reduced in both VS/UWS and MCS relative to controls, mirroring the thalamocortical disconnection seen intracranially [17]. Task-based and resting-state fMRI add triangulation by detecting covert awareness in behaviorally unresponsive patients—now endorsed by both American and European pDOC guidelines [3]. Non-invasive neuromodulation (rTMS, tDCS, peripheral nerve stimulation) has produced modest but significant CRS-R improvements, complementing invasive DBS by showing that thalamocortical circuits can be modulated externally with less anatomical precision [46]. Recent consensus frameworks integrate these modalities into a hierarchical multimodal workflow combining behavioral scales, EEG (including ERPs and TMS-EEG), and functional neuroimaging [2, 47]. This triangulation provides accessible, repeatable assessments deployable where invasive monitoring is not feasible, and validates mechanistic hypotheses from thalamic LFP studies against independent scalp-level and imaging evidence.

#### Limitations of the current evidence base

Several interrelated methodological limitations temper current conclusions about thalamic LFP biomarkers. The intracranial DoC literature consists almost exclusively of small single-center case series or retrospective analyses, with the largest individual-participant meta-analysis encompassing fewer than 80 patients across heterogeneous protocols [34]. Small samples are compounded by marked etiological heterogeneity—traumatic, anoxic, hemorrhagic, and mixed injuries are frequently pooled despite differing pathophysiology—and inconsistent recording conditions including variable electrode targets, stimulation parameters, medication regimens, and time since injury. Evidence maturity also varies across signal domains: spectral power and burst-rate analyses have been replicated across cohorts, whereas aperiodic exponent and CFC measures remain exploratory, with recent work showing aperiodic estimates are themselves sensitive to fitting range and method [22]. Non-invasive approaches face analogous challenges: standardized common data elements for behavioral phenotyping and electrophysiology have only recently been proposed [45, 47], and Curing Coma Campaign documents have consistently identified absent harmonized protocols, shared registries, and adequately powered prospective trials as critical barriers [48–50].

The associative nature of available evidence compels further caution. Correlations between thalamic spectral features and consciousness levels, however neurobiologically plausible, should not be over-interpreted as establishing clinical-grade readiness. Most studies report group-level differences between VS/UWS and MCS rather than individual-level classification accuracy with actionable sensitivity and specificity, and the absence of blinded prospective designs—where LFP predictions are tested against independently adjudicated outcomes—leaves predictive validity unestablished. Narrative reviews like this one also carry inherent selection-bias risks [51], and systematic reviews of neuromodulation in DoC have similarly noted that even the most promising interventions lack the evidence level required for definitive recommendations [36]. The current literature therefore supports a mechanistically informative but preliminary framework requiring large-scale, multicenter, prospective validation before thalamic LFP signatures can become reliable clinical tools.

#### Future directions

Building on these limitations, the most actionable next step is establishing international multicenter intracranial-recording consortia leveraging the standardized common data elements and open-access infrastructure proposed by the Curing Coma Campaign [45, 47–50]. Such consortia would enable prospective enrolment with harmonized electrode targeting, stimulation protocols, and outcome adjudication—prerequisites for adequately powered studies unattainable by isolated centers. Recent network-level analyses identifying optimal thalamic subregions and white-matter pathways associated with consciousness restoration provide anatomically specific hypotheses now ready for prospective testing [18].

A second priority is systematic co-registration of intracranial and extracranial recordings within the same patients—simultaneous thalamic LFP, scalp EEG, TMS-EEG perturbational complexity, and resting-state fMRI—allowing direct calibration of non-invasive biomarkers against their subcortical ground truth. Parallel meta-analytic frameworks synthesizing outcomes across invasive and non-invasive neuromodulation would clarify which circuit-level mechanisms are shared and which are modality-specific [46].

Finally, the field must move toward prospective, preregistered biomarker-validation studies in which LFP-derived spectral and temporal features are tested as diagnostic or prognostic classifiers against independently adjudicated outcomes, with predetermined performance thresholds. Mechanism-guided neuromodulation trials—including adaptive closed-loop DBS paradigms titrating stimulation in real time from electrophysiological feedback—represent a logical translational endpoint, but must be embedded within the ethical safeguards outlined above. Future progress will depend as much on rigorous validation and ethical design as on technical innovation.

## Conclusion

Intracranial recordings from the central thalamus provide a mechanistically privileged but still limited window onto the circuit-level pathophysiology of pDOC. Across the available literature, LFP findings converge in implicating disrupted thalamocortical oscillatory dynamics, impaired functional connectivity, altered aperiodic activity, and reduced nonlinear complexity as correlates of impaired consciousness, with theta-band features particularly informative for both diagnosis and DBS-responsive recovery. These signal domains are best understood not as independent biomarkers but as complementary projections of a shared underlying disturbance in arousal gain, network integration, and dynamical criticality within the anterior forebrain mesocircuit. Direct human evidence remains thin: fewer than a dozen studies have reported intracranial electrophysiology in pDOC, most with small heterogeneous samples and variable recording conditions, and none has simultaneously measured all feature domains in the same cohort. Indirect evidence from anesthesia, non-human primates, and non-invasive neuromodulation enriches mechanistic interpretation but cannot substitute for direct validation in the target population. Bridging this gap will require international multicenter consortia leveraging common data elements and open-access infrastructure, systematic co-registration of intracranial and extracranial modalities, and prospective biomarker-validation trials embedded within rigorous ethical safeguards. The promise of intracranial electrophysiology in pDOC lies not in replacing behavioral or neuroimaging assessment but in complementing them within a multimodal, mechanism-guided framework—one in which the pace of clinical translation will be set as much by methodological rigor and ethical design as by technical innovation.

## Supplementary Information

Additional file 1. Summary of local field potential studies in prolonged disorders of consciousness. This table summarizes patient cohorts, recording targets, electrode types, signal-feature domains analyzed, key findings, and limitations of all studies discussed in the narrative review.

## Data Availability

The data that support the findings of this study are available from the corresponding author, upon reasonable request.

## Abbreviations

ApEn: Approximate entropy
CFC: Cross-frequency coupling
CL: Central lateral (nucleus)
CM-Pf: Centromedian–parafascicular (complex)
CRS-R: Coma Recovery Scale–Revised
DBS: Deep brain stimulation
DoC: Disorders of consciousness (used when referring broadly to consciousness disorders including acute states, or when citing studies that define their cohort as DoC rather than specifically pDOC)
E/I: Excitation–inhibition (balance)
EEG: Electroencephalography/electroencephalogram
fMRI: Functional magnetic resonance imaging
IIT: Integrated Information Theory
LFP(s): Local field potential(s)
MCS: Minimally conscious state
MUA: Multi-unit activity
pDOC: Prolonged disorders of consciousness (the primary focus of this review; refers to chronic consciousness disorders persisting beyond the acute phase)
PCI: Perturbational complexity index
PET: Positron emission tomography
SE: Spectral exponent
STN: Subthalamic nucleus
TCCFC: Thalamocortical cross-correlation function
TMS-EEG: Transcranial magnetic stimulation combined with electroencephalography
VS/UWS: Vegetative state/unresponsive wakefulness syndrome
